# COQ8B nephropathy: Early detection and optimal treatment

**DOI:** 10.1002/mgg3.1360

**Published:** 2020-06-16

**Authors:** Xiaoxiang Song, Xiaoyan Fang, Xiaoshan Tang, Qi Cao, Yihui Zhai, Jing Chen, Jialu Liu, Zhiqing Zhang, Tianchao Xiang, Yanyan Qian, Bingbing Wu, Huijun Wang, Wenhao Zhou, Cuihua Liu, Qian Shen, Hong Xu, Jia Rao

**Affiliations:** ^1^ Department of Nephrology Children's Hospital of Fudan University National Pediatric Medical Center of China Shanghai China; ^2^ Department of Rheumatology and Immunology Children’s Hospital of Soochou University Suzhou China; ^3^ Shanghai Kidney Development and Pediatric Kidney Disease Research Center Shanghai China; ^4^ Shanghai Key Laboratory of Birth Defect Children's Hospital of Fudan University Shanghai China; ^5^ Clinical Genetic Center Children’s Hospital of Fudan University Shanghai China; ^6^ Department of Nephrology and Rheumatology Children's Hospital Affiliated to Zhengzhou University Zheng Zhou China; ^7^ State Key Laboratory of Medical Neurobiology Institutes of Brain Science and School of Basic Medical Science Fudan University Shanghai China

**Keywords:** CoQ10, COQ8B, proteinuria, steroid resistant nephrotic syndrome (SRNS), transplantation

## Abstract

**Background:**

Mutations in *COQ8B* (*615567) as a defect of coenzyme Q10 (CoQ10) cause steroid resistant nephrotic syndrome (SRNS).

**Methods:**

To define the clinical course and prognosis of COQ8B nephropathy, we retrospectively assessed the genotype and phenotype in patients with *COQ8B* mutations from Chinese Children Genetic Kidney Disease Database. We performed the comparing study of renal outcome following CoQ10 treatment and renal transplantation between early genetic detection and delayed genetic detection group.

**Results:**

We identified 20 (5.8%) patients with biallelic mutations of *COQ8B* screening for patients with SRNS, non‐nephrotic proteinuria, or chronic kidney disease (CKD) of unknown origin. Patients with *COQ8B* mutations showed a largely renal‐limited phenotype presenting with proteinuria and/or advanced CKD at the time of diagnosis. Renal biopsy uniformly showed focal segmental glomerulosclerosis. Proteinuria was decreased, whereas the renal function was preserved in five patients following CoQ10 administration combined with angiotensin‐converting enzyme (ACE) inhibitor. The renal survival analysis disclosed a significantly better outcome in early genetic detection group than in delayed genetic detection group (Kaplan–Meier plot and log rank test, *p* = .037). Seven patients underwent deceased donor renal transplantation without recurrence of proteinuria or graft failure. Blood pressure showed decreased significantly during 6 to 12 months post transplantation.

**Conclusions:**

*COQ8B* mutations are one of the most common causes of adolescent‐onset proteinuria and/or CKD of unknown etiology in the Chinese children. Early detection of COQ8B nephropathy following CoQ10 supplementation combined with ACE inhibitor could slow the progression of renal dysfunction. Renal transplantation in patients with *COQ8B* nephropathy showed no recurrence of proteinuria.

## Graphical abstract

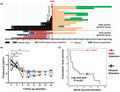

## 1 INTRODUCTION

Proteinuria can be detected in 5% to 10% of children by school urine screening (Murakami, Hayakawa, Yanagihara, & Hukunaga, 2005). Persistent proteinuria was found in 0.1% of them which is an early indication for chronic kidney disease (CKD). Nephrotic syndrome (NS) manifests with significant proteinuria, hypoalbuminemia, and edema. In contrast to other forms of NS, steroid resistant NS (SRNS) does not respond to drug treatment and inevitably progresses to end‐stage renal disease (ESRD), thus, requiring dialysis or renal transplantation for survival (Eddy & Symons, 2003). In SRNS, renal histology reveals focal segmental glomerulosclerosis (FSGS) or diffuse mesangial sclerosis (DMS), which indicate irreversible damage to the glomerulus. Mutations in over 50 genes have been discovered to be monogenic cause of SRNS (Vivante & Hildebrandt, 2016). Recently, recessive mutations in *COQ8B* (Coenzyme Q8B, or *ADCK4,* *615567) have been added to this list as a novel cause of SRNS (Ashraf et al., 2013; Rao et al., 2019).

Coenzyme Q (ubiquinone; CoQ10) is a small lipophilic molecule involved in a series of crucial cellular processes. CoQ10 is an electron shuttle in the mitochondrial respiratory chain, a cofactor of several other mitochondrial dehydrogenases, a modulator of the permeability transition pore, and acts as one of the most important cellular antioxidants. Mutations in several genes encoding enzymes of the CoQ10 biosynthetic pathway (COQ2, *609825; COQ6, *614647; COQ8B, *615567; PDSS2, *610564) are associated with a glomerular phenotype (Heeringa et al., 2011; López et al., 2006; Quinzii et al., 2006). These have been collectively termed CoQ10 glomerulopathy. COQ8B nephropathy is one of the important differential diagnoses in adolescents with SRNS and/or CKD of unknown origin (Atmaca et al., 2017; Korkmaz et al., 2016).

We screened for COQ8B nephropathy among children with SRNS, non‐nephrotic proteinuria, or CKD of unknown origin. By performing whole exon sequencing, we here identified a new cohort of 20 patients with *COQ8B* mutations. Genotype–phenotype analysis, follow‐up of CoQ10 treatment and transplantation revealed the clinical feature and prognosis of the Chinese cohort with COQ8B nephropathy.

## 2 MATERIALS AND METHODS

### 2.1 Study participants and design

The children and adolescents with renal disease aged from birth to 18 years old were recruited from January 1, 2014 to May 31, 2019 by building up a national multicenter registration network (Chinese Children Genetic Kidney Disease Database [CCGKDD], www.ccgkdd.com.cn) (Rao et al., 2019). Following informed consent, we collected pedigree information, clinical and sequence data among the individuals with proteinuria, SRNS, and CKD 3–5 stage with unknown origin. Study approval was obtained from Institutional Review Board (IRB) of Children's Hospital of Fudan University (No. 2018286). Patients with identified mutations of *COQ8B* gene were followed up. Exclusion criteria included (a) the families who refused to perform the genetic test, declined the registration network, or refused the CoQ10 supplementation; or (b) the mean depth coverage of ≥20X or the target coverage region ≥90% was not achieved through quality control (QC) evaluation.

A retrospective analysis for clinical outcome in patients identified *COQ8B* mutations with registration from January 1, 2014 to May 31, 2019. Early genetic detection group (E group) was the patients for whom the gene sequencing was performed before developing into the CKD 5 stage (ESRD). Delayed genetic detection group (D group) was the patients who performed the gene sequencing post ESRD. The follow‐up period started from the initial assessment by the pediatrician, and ended to the occurrence of ESRD or to December 31, 2018, whichever came first.

### 2.2 Genetics

Whole exome sequencing (WES) and variant burden analysis were performed by Wuxi NextCODE and Chigene, respectively. Genomic DNA was isolated from blood lymphocyte and subjected to exome capture using Agilent's SureSelect human all exon kit V5 and NimbleGen technology followed by next generation sequencing on the Illumina HighSeq sequencing platform. Variant interpretation was done by a panel of nephrologists or molecular geneticists with domain expertise in inherited kidney diseases, bioinformaticians, and the clinical molecular geneticists, using the ACMG guidelines for clinical sequence interpretation (Kalia et al., 2017).

### 2.3 Phenotype and clinical outcome

Demographic, initial clinical features including nephrotic proteinuria (24‐hr urine protein ≥1 g) or subnephrotic proteinuria (24‐hr urine protein <1 g), additional phenotype findings including urological system abnormality, heart deficiency, neurological disorder, ocular lesions or hearing impairment, onset age, blood pressure monitored by ambulatory blood pressure monitoring (ABPM), and renal function were recorded.

Proteinuria was monitored by the urine protein/creatinine ratio (Up/c, mmol/mmol) of a morning‐void (Spot) specimen. A standard methodology was utilized: ABPM for wake hours measurements were performed every 20 min; for sleep hours, every 60 min (Welch Allyn ABPM 6100). In order to improve the quality of the BP recordings, patients were encouraged to maintain their usual activities and to complete a diary of events over 24 hr. ABPM parameters included mean systolic and diastolic BP at daytime, nighttime, and 24 hr; mean arterial pressure (MAP) percentiles and Z scores calculated for sex and height according to the reference data in healthy central European pediatric population (Flynn et al., 2014; Yip et al., 2014). Postrenal transplantation, the urine protein, blood pressure by ABPM, and creatinine serum at least level every 3 months were also recorded.

Comparison study was performed for the patients identified recessive mutations of *COQ8B* to address the following hypothesis: early diagnosis of COQ8B nephropathy will be medically beneficial with the CoQ10 supplementation. The start of the follow‐up period was taken as the date of disease onset. The primary end point was progression to ESRD (eGFR < 15 ml/min/1.73 m^2^). The secondary end point was progression to CKD 3–4 stage (eGFR 15–30 ml/min/1.73 m^2^). All the end points were recorded by review of patients’ electronic and hard copy medical files and renal function estimated by adjusted *Schwartz formula* (Levey et al., 2003; Stevens & Levin, 2013).

### 2.4 CoQ10 supplementation

Supplementation of CoQ10 was initiated in individuals identified with recessive mutations of *COQ8B* following consent. As there were no consensus on dosage, CoQ10 supplementation was administrated with empirical doses between 15 and 30 mg kg^−1^ day^−1^ in two or three divided doses. Fosinopril, one of the angiotensin‐converting enzyme (ACE) inhibitor was administrated at the same time with the dose of 0.25–0.5 mg kg^−1^ day^−1^ according to the KDOQI guidelines for the effects on proteinuria and renal function (Stevens & Levin, 2013).

### 2.5 Ambulatory blood pressure control in children post–kidney transplantation

For patients underwent renal transplantation, ABPM was routinely conducted during the period of pretransplant (pre‐Tx), 0–3 months post‐transplant (post‐Tx0–3mon), 3–6 months posttransplant (post‐Tx3–6mon), 6–12 months posttransplant (post‐Tx6–12mon), 12–24 months posttransplant (post‐Tx12–24mon), 24–36 months posttransplant (post‐Tx24–36mon), and 36–48 months posttransplant (post‐Tx36–48mon). The Z score of MAP was analyzed between the different period posttransplantation. During the same phase from 2014 to 2018, a total 59 pediatric patients underwent renal transplantation who were followed up in our center. And comparing study was performed between patients of COQ8B nephropathy and patient group of the other 52 patients with the other kind of renal disease (non‐COQ8B nephropathy) posttransplantation in the same study phase.

### 2.6 Statistics

Data were analyzed using Excel. Continuous variables were summarized with median, IQR and categorical data were summarized with proportions. Quantitative value was compared by Mann–Whitney test and the frequencies were compared using the Fisher exact probability test. Kaplan–Meier analysis and the log rank test were performed to compare the renal survival between the early detection group and the delayed detection group with SPSS software, version 22 (IBM). We compared the Z value of MAP for 24‐hr, daytime and nighttime during the different follow‐up phase by Wilcoxon matched‐pairs single rank test. Figures have been performed using GraphPad Prism 7.0.

## 3 RESULTS

### 3.1 Mutations in COQ8B glomerulopathy

**FIGURE 1:**
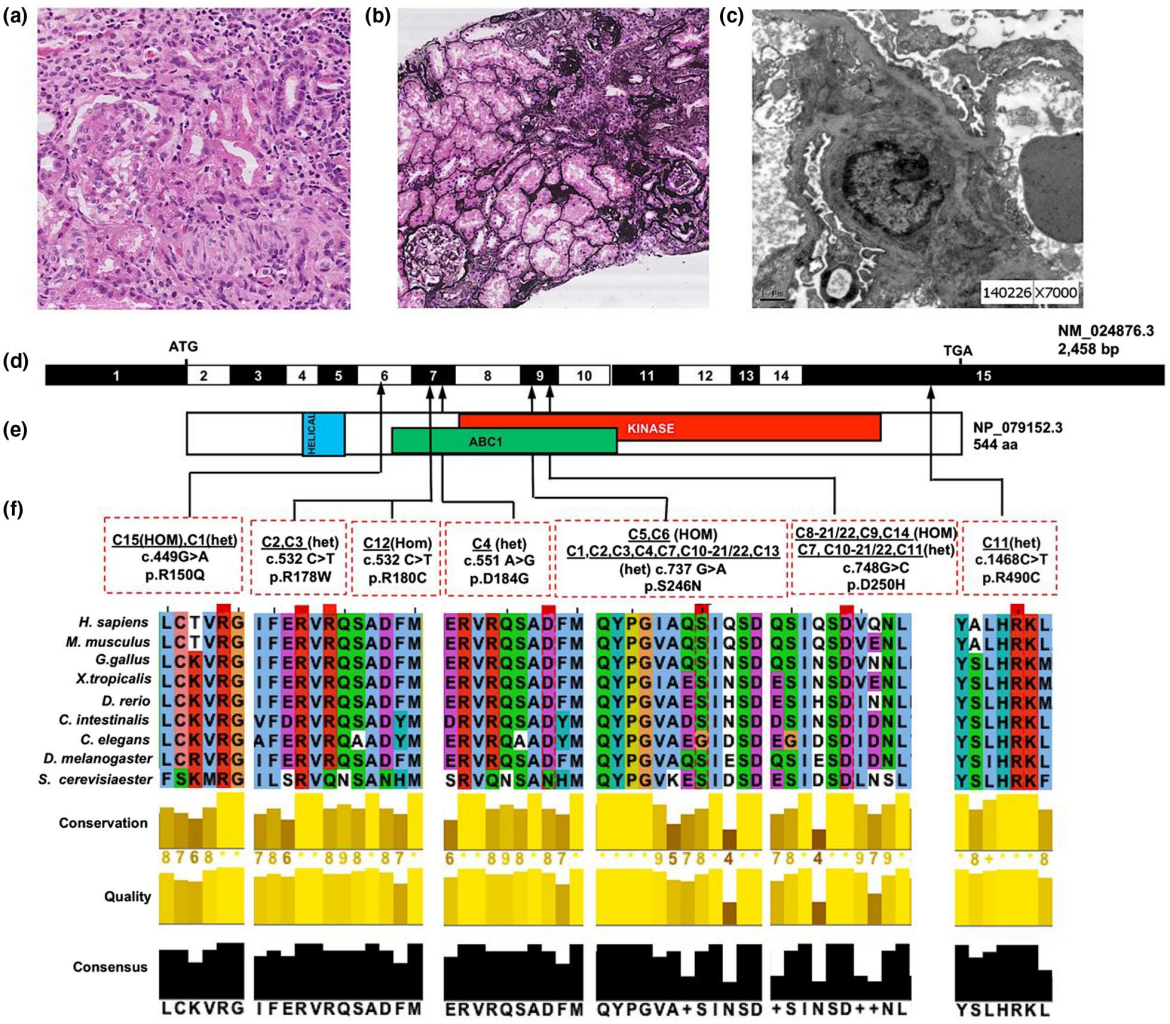
Exon capture and massively parallel sequencing reveal *COQ8B* mutations as causing SRNS and CKD. (a) Renal histology of individual C9 reveals FSGS by HE staining. (b) Renal histology of individual C9 shows FSGS and global glomerulosclerosis by Silver Jones Methenamine staining. (c) Renal histology of individual C9 shows foot process effacement. (d) Exon structure of human *COQ8B* cDNA. The *COQ8B* gene contains 15 exons. Positions of start codon (ATG) and of stop codon (TGA) are indicated. For the mutations detected (see f) arrows indicate positions in relation to exons and protein domains (see e). (e) Domain structure of the COQ8B protein. Extent of predicted domains, helical, ABC1, and kinase is depicted by colored bars, in relation to encoding exon position. (f) Nine different *COQ8B* mutations in 17 families with SRNS. Nucleotide change and amino acid changes (see Table S1) are given above sequence traces. Arrow heads denote altered nucleotides. Lines and arrows indicate positions of mutations in relation to exons (see d) and protein domains (see e). For the missense mutations, conservation across evolution of altered amino acid residues is shown. CKD, chronic kidney disease; FSGS, focal segmental glomerulosclerosis

Screening the disease causative genes by WES in 345 patients with SRNS, non‐nephrotic proteinuria, or CKD 3–5 stage on unknown origin, we identified 20 patients (5.8%) from 17 families with biallelic mutations of *COQ8B* (Figure 1, Table 1). Mutation analysis revealed three novel sequence variants and three previously reported recessive mutations in *COQ8B*. Two mutations, namely c.737G>A (p.S246N) and c.748G>A (p.D250H), were the most prevalent in our cohort. The recurrent variant c.737G>A (p.S246N) was reported in 10 unrelated families originating from the same region in the central‐east of China, suggesting a putative founder effect. The recurrent variant c.748G>A (p.D250H) was found in two siblings (C8‐21 and C8‐22) from a consanguineous family with homozygous mutation and another seven unrelated families originating from the same region in the east of China, also suggesting a founder effect. Segregation and bioinformatic information on the variants are provided in Table S1.

**TABLE 1:** The clinical feature of the patients with COQ8B nephropathy

	Early detection	Delayed detection	*p* value
Patients (*n*)	5	15	
Female: Male	2:3	7:8	
Age onset (years)	5.0 (2.5, 8.0)	9.0 (3.0, 11.0)	.07
Initial proteinuria (Up/Cr mmol/mmol)	4.1 (3.3, 5.1)	6.8 (4.0, 7.8)	.26
Initial eGFR (ml/min/1.73 m^2^)	115.0 (102.3, 118.0)	110 (35.0, 118.0)	.25
Age of genetic diagnosis (years)	5.5 (3.1, 9.6)	10.9 (7.7, 12.2)	.10
Time from genetic diagnosis until end of follow‐up (years)	4.0 (3.0, 6.5)	4.5 (2.0, 6.0)	.73

Note:

Proteinuria was monitored by the urine protein/creatinine ratio (Up/c, mmol/mmol) of a morning‐void (Spot) specimen. Renal function was evaluated by estimated GFR (*Schwartz formula eGFR* = KL/Scr, with K = 0.49 in CKD1‐2, K = 0.36 in CKD3‐5). Data were shown as median with interquartile range (IQR). Quantitative value was compared by Mann–Whitney test indicated by *p* value.

### 3.2 Phenotype of COQ8B nephropathy

Between 01/2014 and 05/2018, we enrolled 20 patients with COQ8B nephropathy from the CCGKDD. All the twenty patients had recessive mutations of *COQ8B* and completed the follow‐up. Initial diagnosis of renal disease was performed at a median age of 7.0 (IQR 3.0–10.0) years in the 20 individuals. Consanguinity was present in four (23.5%) families. Patients with *COQ8B* mutations were initially manifested in adolescence, with no cases manifesting before 2 years of age, and they all had proteinuria. They manifested typically with mild to moderate proteinuria screened by school urine test or random urine test in eight patients. Microscopic hematuria was also detected in three patients. Six patients were initially diagnosed with eGFR below 30 ml/min/1.73 m^2^. Advanced CKD was present in seven patients at the time of diagnosis. The seven patients progressed to ESRD occurred within a median of 11.0 years old (IQR 7.8–13.2). Renal biopsy revealed FSGS in nine cases, mesangial proliferation glomerulonephritis in four cases, and DMS in one case. Six had not been subjected to biopsy. (Table S1).

Additional phenotype findings included seizure (2), vesicoureteral reflux (1), multicystic dysplastic kidney (1), renal calcinosis (1), retinitis (1), cataract (1), and ovarian cyst (1). Short stature was shown in five cases associated with prolonged daily prednisone treatment or CKD. No histories of hearing problems, cardiomyopathy, muscle weakness, optical nerve atrophy, or hematologic or endocrinologic abnormalities were reported in any patient. Hypertension was more common in patients COQ8B nephropathy. Two cases with *COQ8B* mutations were diagnosed seizures of hypertension‐related reversible posterior encephalopathy.

**FIGURE 2:**
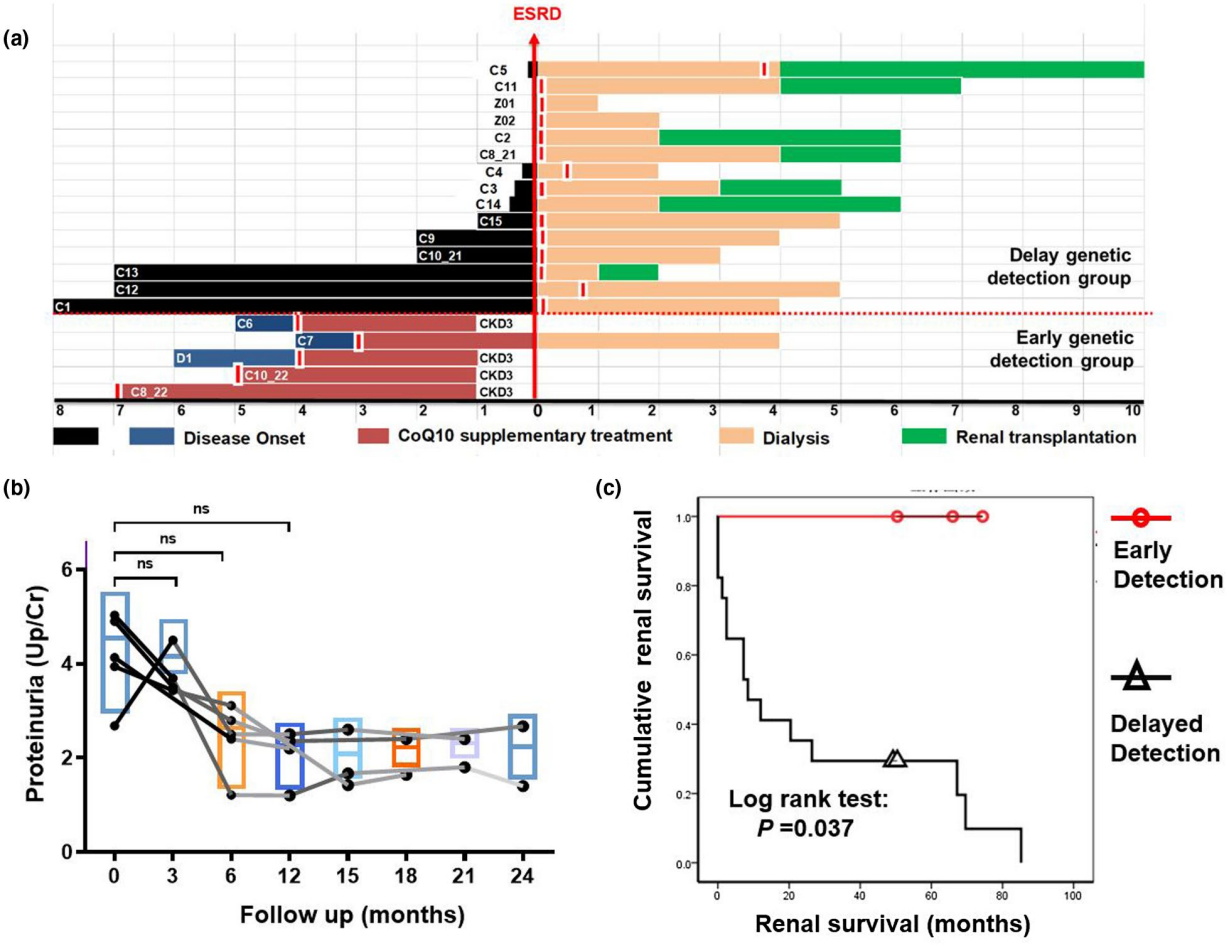
Renal outcome in COQ8B nephropathy. (a) Relationship between the disease onset and the time point at which WES performed (red vertical hatch) and relevant treatment events in 20 cases of COQ8B nephropathy. For each patient, the treatment of CoQ10 (red bar), the primary endpoint of end stage renal disease (ESRD, X), peritoneal dialysis (orange bar) and renal transplantation (green bar) were depicted. About 15 patients completed the genetic testing just before developing into ESRD or post dialysis. Five cases had been diagnosed of COQ8B nephropathy during an asymptomatic proteinuria period through urinary screening for siblings from the affected family, occasional urine test or urine screening in school. (b) Urinary protein level over the time of following CoQ10 initiation in five individuals genetic diagnosed with *COQ8B* mutation. After a median follow‐up duration of 21.0 (range from 12 to 24) months following CoQ10 administration, proteinuria decreased. The median Up/cre following CoQ10 supplementation and ACE inhibitor (Median 2.4; IQR 1.7–3.0; range 1.2–4.5) was significantly lower than that before CoQ10 treatment (Median 4.1; IQR 3.3–5.1; range 2.8–23.7) by Mann–Whitney test (*p* = .04). There was no significant difference of the proteinuria because the small sample size by Wilcoxon matched‐pairs single rank test. (c) Renal survival curve of patients with COQ8B nephropathy in comparison in the early detection group (*n* = 5, red) and the delayed detection group (*n* = 15, black). Whereas 15 cases in the delayed detection group had developed into ESRD (median interval from disease onset to ESRD was 7.8 months), only one case in the early detection group progressed into ESRD, which is significant (*p* = .043). ESRD, end‐stage renal disease

Five patients of early detection group and fifteen patients of delayed detection group were enrolled in the study (Figure 2, Table 1). For the characteristics at disease onset, there was no significant difference in proteinuria and renal function initially (*p* > .05). There was no significant difference in age of disease onset or age performing genetic test (*p* > .05, Table 1).

### 3.3 Response to CoQ_10_ treatment

Here, we evaluated the efficacy of CoQ10 supplementation in five patients who had been diagnosed during an asymptomatic proteinuria period through urinary screening for siblings from the affected family, occasional urine test or urine screening in school. In these five patients, median eGFR just before CoQ10 administration was 115.0 ml/min/1.73 m^2^ (IQR 102.3–1118.05) and the ratio of urinary protein and creatinine (Up/cre) was 4.1 (IQR 3.3–5.1). After a median follow‐up duration of 21.0 (range from 12 to 24) months following CoQ10 administration, proteinuria decreased (median Up/cre 2.4, IQR 1.7–3.0；Figure 2). Unfortunately, there was no significant difference of the proteinuria because the small sample size. At the end of the study, one patient at the age of 13.2 years old developed into ESRD, and the other four patients progressed to CKD 3 stage (Figure 2a,b). No side effects was observed in the patients who received CoQ10.

### 3.4 Renal outcome in COQ8B nephropathy

There was no significant difference in the initial clinical features including age of onset, age of genetic diagnosis, proteinuria or eGFR between patients from early genetic detection group and patients from delayed genetic detection group (*p* > .05 respectively by *Mann–Whitney test*, Table 1.) The renal survival curve of the patients with COQ8B nephropathy was analyzed. The median interval from genetic diagnosis till the study end was 5.5 (range 3.0–7.0) years in early detection group and 4.5 (range 1.0–7.0) years (Figure 2, Table 1). The renal survival analysis using a Kaplan–Meier plot and log rank test of the end point of ESRD was performed and disclosed a significantly better outcome (*p* = .037) in early detection group than in delayed detection group (Figure 2c).

### 3.5 Follow up post kidney transplantation

**FIGURE 3:**
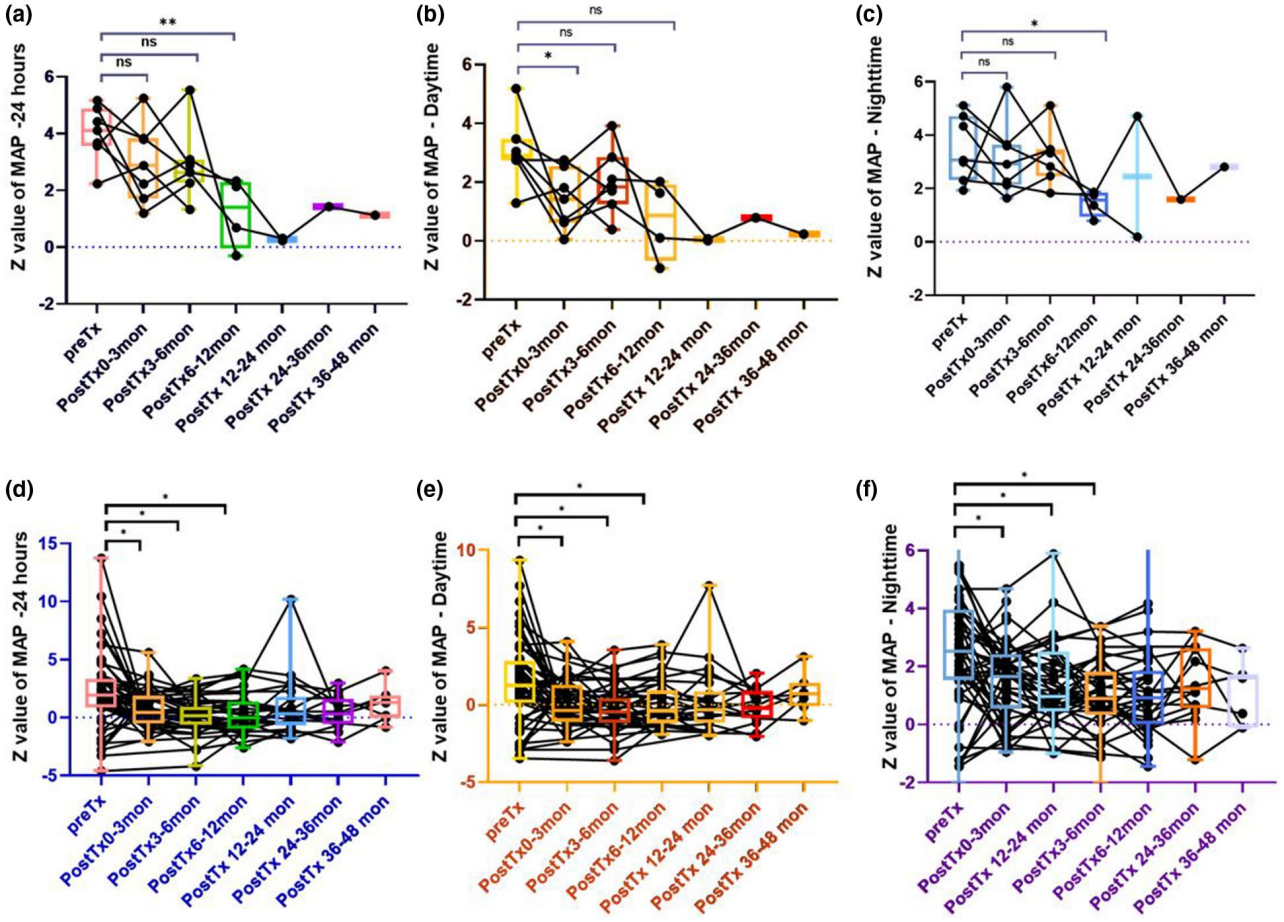
Follow‐up of Ambulatory blood pressure monitoring (ABPM) in the 7 patients with COQ8B glomerulopathy post kidney transplantation (a–c) and the 52 patients with non‐COQ8B glomerulopathy post kidney transplantation (d–f). Comparing the Z value of mean arterial pressure (MAP) for 24‐hr, daytime and nighttime during the period of pre‐transplant (pre‐Tx), 0–3 months posttransplant (post‐Tx0–3mon), 3–6 months posttransplant (post‐Tx3–6mon), 6–12 months posttransplant (post‐Tx6–12mon), 12–24 months posttransplant (post‐Tx12–24mon), 24–36 months posttransplant (post‐Tx24–36mon), and 36–48 months posttransplant (post‐Tx36–48mon) by *Wilcoxon matched‐pairs single rank test*. (a) Analysis Z value ofMAP for 24‐hr. There was no significant difference between the Z value of pre‐Tx and that of post‐Tx0–3mon or post‐Tx3–6mon (*p* = .297. *p* = .125). It was significant lower for the Z value of post‐Tx6–12mon compared with that of pre‐Tx (*p = .031). (b) Analysis Z value of MAP for daytime. It was significant lower for the Z value of post‐Tx0–3mon compared with that of pre‐Tx (*p = .0466.). It showed no significant difference for the Z value of post‐Tx3–6mon, or post‐Tx6–12mon compared with that of pre‐Tx (*p* = .062, *p* = .125). (c) Analysis Z value of mean arterial pressure (MAP) for nighttime. There was no significant difference between the Z value of pre‐Tx and that of post‐Tx0–3mon or that of post‐Tx3–6mon (*p* = .297; *p* = .438). It was significant lower for the Z value of post‐Tx6–12mon, or post‐Tx6–12mon compared with that of pre‐Tx (*p = .041). (d) Analysis Z value of MAPfor 24‐hr. It was significant lower for the Z value of that of post‐Tx0–3mon/post‐Tx3–6mon/post‐Tx6–12mon compared with that of pre‐Tx (*p = .0005, .0007, .0166). (e) Analysis Z value of MAP for daytime. It was significant lower for the Z value of post‐Tx0–3mon/post‐Tx3–6mon/post‐Tx6–12mon compared with that of pre‐Tx (*p = .0006, .0004, .0274). (f) Analysis Z value of mean arterial pressure (MAP) for nighttime. It was significant lower for the Z value of post‐Tx0–3mon/post‐Tx3–6mon/post‐Tx6–12mon compared with that of pre‐Tx (*p = .0008, .0002, .0053)

Seven patients underwent deceased donor renal transplantation at a median age of 9.6 (IQR, 8.4–12.3) years old. The median follow‐up duration of the time of study was 17.8 (IQR, 15.2–19.7; range 11.0–60.6) months. Two of them had initial pathological diagnosis of FSGS, two of mesangial proliferation glomerulonephritis and three without biopsy. All of them had excellent graft survival including two cases who developed acute rejection without graft failure. There was no recurrence of NS or proteinuria in all the seven patients. The predominant immunosuppressive regimen was tacrolimus and mycophenolate mofetil. Prednisone or methylprednisolone was administrated for acute rejection in short‐term. Cardiovascular complication was assessed mainly by ABPM. Analysis of the Z value of MAP showed Z values of MAP‐24‐hr had been significant lower during the period of 6–12 months posttransplant compared with that of pretransplant (*p* = .031). Interestingly, the Z value of MAP‐Daytime was significant lower at 3 months posttransplant compared with pretransplant (*p* = .047). While it showed no significant difference in the period of 3–12 months posttransplant (*p* = .297; 0.438). For the Z value of MAP‐Nighttime, there was no significant difference between that of 1 to 6 months posttransplant (*p* = .297; 0.438). But, the Z value of MAP‐Nighttime showed significant lower at 6–12 months post‐transplant compared with pretransplant (*p* = .041). Analysis of the Z value of MAP in the other 52 patients with non‐COQ8B nephropathy showed controlled hypertension posttransplantation (Figure 3).

## 4 DISCUSSION

Our data indicated a 5.8% prevalence of recessive mutations in *COQ8B* gene in patients with SRNS, non‐nephrotic proteinuria, or CKD on unknown origin. Our COQ8B nephropathy cohort presented the distinct mutation spectrum of *COQ8B* in Chinese patients. Two mutations, namely p.S246N and p.D250H, were the most prevalent.

We described the clinical features which make the COQ8B nephropathy unique to the congenital glomerulopathies. The renal phenotype of COQ8B nephropathy was characterized by adolescence onset with mild to moderate proteinuria and absence of hematuria or edema in the majority of cases. As a consequence of the asymptomatic early course, advanced CKD was often presented at the time of diagnosis. Hypertension was more common to patients COQ8B nephropathy. Sequence variation in *COQ8B* (5.7%), *WT1* (*607102, 5.4%), and *NPHS1* (*602716, 2.8%) were the most frequently found in SRNS group as shown in the CCGKDD (Rao et al., 2019). COQ8B nephropathy typically manifests as an isolated nephropathy with only occasional extrarenal symptoms. In our cohort, 12 individuals never showed any extrarenal system involvement. Two patients presented seizures of hypertension‐related reversible posterior encephalopathy. Two cases of congenital anomalies of the kidney and urinary tract (CAKUT) phenotype and one case with renal calcinosis, one case with retinitis and one case with cataract were reported.

Early detection of COQ8B nephropathy was achieved by urine screening for proteinuria. There were five patients diagnosed during asymptomatic proteinuria by screening for siblings from the affected families, occasional urinary test or urine screening in school. Successful CoQ_10_ treatment of SRNS had been described previously in individuals with *COQ8B* mutation (Atmaca et al., 2017; Korkmaz et al., 2016). Hence, we had the opportunity to initiate CoQ10 treatment for the five patients before renal failure. Our findings demonstrated the beneficial effect of early CoQ10 supplementation combined with ACEI treatment of proteinuria. At the end of the study, the eldest patient with CoQ_10_ treatment was 13.2 years old developing into ESRD and the other four cases developing into CKD 3 stage. Considering the median age of 11 for progression to ESRD in our cohort, we must monitor the renal function carefully for this adolescent patient. The renal survival showed the significantly better outcome in early detection group than in delayed detection group. However, the current study was too short to predict the final outcome. Long‐term follow‐up for renal function and proteinuria should be carried out.

Renal transplantation was reported here in seven pediatric patients with COQ8B nephropathy. FSGS is known to be the leading cause of recurrent NS post transplantation (Patrakka et al., 2002). It was reported that recurrence rate was low (but not zero) in the genetic forms of FSGS such as *NPHS2* (*604766), *NPHS1* or *COL4A5* (*303630) mutations (Jungraithmayr et al., 2011; Levey et al., 2003; Patrakka et al., 2002). None of the former studies presented the outcome of transplantation in the patients with COQ8B mutation. In the present study, there was no recurrence of NS or proteinuria in all the seven patients even with two cases of FSGS and two cases with mesangial proliferation glomerulonephritis. All of them had excellent graft survival postdeceased donor renal transplantation with the median follow‐up duration of 17.8 months. However, there was no case of living related donor (LRD) in this study. We should consider genetic screening for genetic mutation including *COQ8B* in LRD. Because, the COQ8B nephropathy is recessive we would accept a heterozygous donor.

Hypertension is one of the most common clinical problems seen in kidney transplant recipients and is associated with shortened allograft survival and increased cardiovascular (CV) morbidity and mortality (Mange, Cizman, Joffe, & Feldman, 2000). A longitudinal study of ABP in pediatric and young adult kidney transplant recipients demonstrated improved control of BP over time for patients followed by ABPM (Hamdani et al., 2017). However, the prevalence of masked uncontrolled hypertension remained relatively high (Mange et al., 2000). We underwent the ABPM for the seven patients posttransplantation. As MAP has been shown to be very helpful in detecting mild hypertension in patients with normal/borderline systolic BP and diastolic BP (Suláková & Feber, 2013), the Z value for MAP was evaluated this study. Lower blood pressure was achieved during the period of 6 to 12 months posttransplantation as indicated by MAP‐24‐hr and MAP‐nighttime. While the MAP‐daytime was not different between the period of 3 to 12 months and pretransplantation. Previous studies indicated that clinic BP levels one year after transplantation predict future graft function, even when corrected for GFR (Hamdani et al., 2017). A large cohort of 123 children and young adults following kidney transplantation (median follow‐up time 2.3 years) showed improvements in many ABP parameters, including mean 24‐hr SBP and DBP indices, mean wake SBP index, and mean sleep SBP and DBP indices (Hamdani et al., 2017). Based on the fundamental role of CoQ10 in mitochondrial bioenergetics and its well‐acknowledged antioxidant properties, several clinical trials evaluating CoQ10 have been undertaken in cardiovascular disorders of aging including chronic heart failure, hypertension, and endothelial dysfunction (Ho, Li, & Wright, 2016; Tran, Mitchell, Kennedy, & Giles, 2001). Blood pressure control should pay more attention to the patients with COQ8B nephropathy posttransplantation. We recommended CoQ10 supplementation accompanied with immunosuppressants for these patients.

## 5 CONCLUSIONS

COQ8B nephropathy is a genetic cause of adolescent onset proteinuria or SRNS. This recessive Mendelian disease often manifests as isolated renal phenotype. Despite the late clinical manifestation, rapid progression to ESRD is common. Early detection of COQ8B nephropathy depended on urine screening and genetic screening. It will help to identify the pediatric patients who could benefit from the CoQ10 supplementation. Patients with *COQ8B* mutations underwent renal transplantation without recurrence. Long‐term follow‐up of blood pressure, proteinuria, and renal function should be carried out for the cohort with COQ8B nephropathy.

## CONFLICTS OF INTEREST

All authors have nothing to declare.

## AUTHORS’ CONTRIBUTION

JR. and HX: designed and supervised the study, wrote the manuscript. XXS and XYF performed clinical examinations, collected blood samples, wrote the clinical part of the manuscript. YYQ, BBW, HJW: performed analysis of whole exome sequencing data; JC, YHZ, QC, JLL, ZWZ, CHL: clinical follow‐up and collect the clinical information; JR, HX, WHZ critically revised the manuscript.

## COMPLIANCE WITH ETHICAL STANDARDS

### Ethical approval

All procedure performed in studies involving human participants were in accordance with the ethical standards of the Ethical Committee for scientific research approval of the Children's Hospital of Fudan University and with the 1964 Helsinki declaration and its later amendments or comparable ethical standards. The clinical and research activities being reported are consistent with the Principles of the Declaration of Istanbul as outlined in the “Declaration of Istanbul on Organ Trafficking and Transplant Tourism.”

### Informed consent

Written informed consent was obtained from all patients and their parents for publication.

## Supporting information

Table S1

## Data Availability

The data that support the findings of this study are available from the corresponding author upon reasonable request.
