# Supplementary material for: COQ8B nephropathy: Early detection and optimal treatment
Source: Mol Genet Genomic Med. 2020 Jun 16;8(8):e1360. doi: 10.1002/mgg3.1360 (PMC7434746; doi:10.1002/mgg3.1360)
Supplement: Supplementary file 1 — Table S1 [file MGG3-8-e1360-s001.docx]

**Table S1: Mutations and clinical follow-up of 20 patients with mutations in the *COQ8B* (*ADCK4)* gene**

| **Patient**  **ID** | **Nucleotide change**  **(ENST00000324464)** | **Amino acid change** | **Exon (Zygosity, Segregation)** | **SIFT** | **MT** | **Amino acid conservation to species** | **gnomAD** | **Gender** | **Parental Consanguinity** | **Age of**  **onset**  **(years)** | **Initial**  **presentation** | **Renal Biopsy** | **Age of ESRD**  **(years)** | **Extrarenal involvement** | **Treatment** |
| --- | --- | --- | --- | --- | --- | --- | --- | --- | --- | --- | --- | --- | --- | --- | --- |
| C15 | c.449G>A | p.R150Q | Exon6 (het,m，het,p) | Del | DC | *S. cerevisiaester* | C=3/T=118386 | F | Yes | 10 | Proteinuria | N.D. | 11 | VUR | PD |
| C1 | c.449G>A  c.737G>A | p.R150Q  p. S246N | Exon6 (het,m)  Exon 9 (het,p) | Del  Del | DC  DC | *S. cerevisiaester*  *C. intestinalis* | C=3/T=118386  C=11/T=  119666 | M | No | 2.4 | Proteinuria | N.D. | 8 | None | PD |
| C2 | c.532C>T  c.737G>A | p.R178W  p.S246N | Exon7 (het,p)  Exon 9 (het,m) | Del  Del | DC  DC | *S. cerevisiaester*  *C. intestinalis* | C=1/T=96832  C=11/T=  119666 | M | No | 6.5 | Proteinuria  CKD5 | Diffuse mesangial sclerosis | 6.5 | None | PD-Tx |
| C3 | c.532C>T  c.737G>A | p.R178W  p.S246N | Exon7 (het,p)  Exon 9 (het,m) | Del  Del | DC  DC | *S. cerevisiaester*  *C. intestinalis* | C=1/T=96832  C=11/T=119666 | M | No | 10.2 | Proteinuria  CKD5 | None | 10.9 | Macula Retinitis | PD-Tx |
| C12 | c.538C>T | p.R180C | Exon7  (Hom)  (m,het;p,het) | Del | DC | *S. cerevisiaester* | None | F | Yes | 4.4 | Proteinuria  CKD5 | FSGS | 11.5 | Ovarian cyst | no response to to steroid or FKFK506  PD |
| C4 | c.551A>G  c.737G>A | p.D184G  p.S246N | Exon7 (het,p)  Exon 9 (het,m) | Del  Del | DC  DC | *D. melanogaster*  *C. intestinalis* | None  C=11/T=119666 | M | no | 2.9 | Proteinuria  CKD5 | FSGS | 3.1 | None | PD |
| C5 | c.737G>A | p.S246N | Exon 9 (HOM)  m(het), p(het) | Del | DC | *D. melanogaster* | C=11/T=119666 | F | No | 7.6 | SRNS | FSGS | 7.7 | None | PD  Tx |
| C6 | c.737G>A | p.S246N | Exon 9 (HOM)  m(het), p(het) | Del | DC |  | C=11/T=119666 | F | No | 3.1 | SRNS | FSGS | None | None | no response to steroids, Treat with ACEI, CoQ10 |
| C7 | c.737G>A  c.748G>C | p.S246N  p.D250H | Exon7 (het,m)  Exon 9 (het,p) | Del  Del | DC  DC | *D.*  *melanogaster*  *S. cerevisiaester* | C=11/T=119666  C=3/G=119350 | F | No | 9.3 | Proteinuria | FSGS | 13.5 | None | no response to steroids  Treat with ACEI, CoQ10  PD |
| D1 | c.737G>A  c.748G>C | p.S246N  p.D250H | Exon7 (het,p)  Exon 9 (het,m) | Del  Del | DC  DC | *D.*  *melanogaster*  *S. cerevisiaester* | C=11/T=119666  C=3/G=119350 | M | No | 7.0 | SRNS | mesangial proliferation glomerulonephritis | - | Renal calcinosis, short stature | no response to steroids  Treat with ACEI, CoQ10 |
| C13 | c.737G>A  c.936-938  delGGT | p.S246N  p.V313del | Exon7 (het,m)  Exon 32 (het,p) | Del  - | DC  - | *D. melanogaster*  *-* | C=11/T=119666  None | F | No | 3.6 | Proteinuria | FSGS | 9.4 | None | no response to steroids or Tac  PD-Tx |
| C8-21 | c.748G>C | p.D250H | Exon 9 (HOM)  m(het), p(het) | Del | DC | *S. cerevisiaester* | C=3/G=119350 | F | Yes | 9.8 | SRNS  CKD5 | mesangial proliferation glomerulonephritis | 9.6 | seizure | no response to steroids, Tac；  PD-Tx |
| C8-22 | c.748G>C | p.D250H | Exon 9 (HOM)  m(het), p(het) | Del | DC | *S. cerevisiaester* | C=3/G=119350 | M | Yes | 2.0 | SRNS | mesangial proliferation glomerulonephritis | None | Cataract | no response to steroids or Tac;  Treat with ACEI, CoQ10 |
| C14 | c.748G>C | p.D250H | Exon 9 (HOM)  m(het), p(het) | Del | DC | *S. cerevisiaester* | C=3/G=119350 | M | Yes | 3.3 | SRNS | N.D. | 3.9 | arrhythmia | no response to steroids or Tac;  Treat with ACEI, CoQ10  PD Tx |
| C10-21 | c.748G>C | p.D250H | Exon 9 (HOM)  m(het), p(het) | Del | DC | *S. cerevisiaester* | C=3/G=119350 | F | NO | 10.5 | Proteinuria  CKD4 | None | 12.2 | None | Treat with CoQ10  PD |
| C10-22 | c.748G>C | p.D250H | Exon 9 (HOM)  m(het), p(het) | Del | DC | *S. cerevisiaester* | C=3/G=119350 | M | No | 5.3 | Proteinuria | FSGS | None | None | Treat with ACEI, CoQ10 |
| C9 | c.748G>C | p.D250H | Exon 9 (HOM)  m(het), p(het) | Del | DC | *S. cerevisiaester* | C=3/G=119350 | F | No | 11.7 | Proteinuria | mesangial proliferation glomerulonephritis | 13.9 | Multicystic Dysplastic Kidney on the right side | Treat with ACEI, CoQ10  PD |
| C11 | c.748G>C  c.1468C>T | p.D250H  p.R490C | Exon 9 (het,p)  Exon 15  (het, de novo) | Del  Del | DC  DC | *S. cerevisiaester*  *S. cerevisiaester* | C=3/G=119350  G=2/A=65552 | F | No | 11.1 | Proteinuria | N.D. | 11.1 | None  Seizure | PD-TX |
| Z01 | c.893+2T>A  c.1035+3A>G | -  - | Intron 9 (het,m)  Intron 10  (het, de novo) | Splice;  splice | - | *-* | A=3/T=250162 (A=3/T=18380 in East Asia)  T=11/G=281218 (none in Asian) | M | No | 15 | Proteinuria | FSGS | 17.2 | None | HD |
| Z02 | c.770+2T>A  c.912+3A>G | -  - | Intron 9 (het,m)  Intron 10  (het) | Splice;  splice | - | *-* | A=3/T=250162 (A=3/T=18380 in East Asia)  T=11/G=281218 (none in Asian) | M | No | 16 | Proteinuria | N.D. | 17.6 | None | HD |

HOM, homozygous in affected individual; het, heterozygous in affected individual; M, heterozygous mutation identified in mother; P, heterozygous mutation identified in father; SIFT, Sortig Intolerant From Tolerant; MT, MutationTaster; DC, disease causing gnomAD,Genome, Aggregation Database; ND, no data or DNA available; SRNS, steroid-resistant nephrotic syndrome; ESRD, end stage renal disease; FSGS, focal segmental glomerulosclerosis; ACE-I, ACE inhibitor; CoQ10, treatment with CoQ10; PD, peritoneal dialysis; HD, hemodialysis; Tx, transplantation
